# Impact of the Chronic Ischemic Stroke Microenvironment on Silk Fibroin Hydrogel Biodegradation and *De Novo* Tissue Formation

**DOI:** 10.1021/acsomega.6c00820

**Published:** 2026-03-28

**Authors:** Suttinee Phuagkhaopong, Natalia Gorenkova, Panicha Aruvornlop, Hilary V. O. Carswell, F. Philipp Seib

**Affiliations:** 1 Strathclyde Institute of Pharmacy and Biomedical Sciences, 150721 University of Strathclyde, Glasgow G4 ORE, United Kingdom; 2 Department of Pharmacology, Faculty of Medicine, 26683 Chulalongkorn University, Bangkok 10330, Thailand; 3 Institute of Pharmacy, Department of Pharmaceutical Technology and Biopharmaceutics, 9378 Friedrich Schiller University Jena, Lessingstr. 8, Jena 07743, Germany; 4 Branch Bioresources, Fraunhofer Institute for Molecular Biology and Applied Ecology, Ohlebergsweg 12, Giessen 35392, Germany

## Abstract

The brain has limited spontaneous tissue regeneration capacity after stroke, partly due to the absence of an extracellular matrix in the stroke microenvironment. Self-assembling silk fibroin hydrogels can serve as a tissue-mimetic extracellular matrix; however, more information is needed on their behavior in the chronic stroke setting. We hypothesized that in the chronic stroke setting, self-assembling silk fibroin hydrogels serve as a reliable support matrix for regeneration in the stroke cavity. In this study, male Sprague–Dawley rats (240–290 g, 8–9 weeks old (*n* = 8) underwent transient middle cerebral artery occlusion 2 weeks before stereotactic injection of 4% w/v self-assembling silk fibroin hydrogels into the stroke cavity. Animals were randomly assigned to be terminated at 6– and 12–months postimplantation (*n* = 4/group) for blinded immunohistological analysis of the in situ distribution of the silk hydrogels and cellular infiltration and characterization. Results showed that robust *in situ* gelation with a good hydrogel–host tissue interface was observed with hydrogel remnants still evident at 1-year postgrafting. At 6 months postgrafting, most cells—primarily astrocytes and microglia/macrophages—were localized at the tissue–hydrogel interface and were CD206+ expressing, whereas the cells that substantially infiltrated the center of the hydrogels at 12 months showed a hybrid of CD86+ and CD206+ phenotypes. The hydrogel areas surrounded by macrophages showed evidence of degradation, potentially providing a niche for endogenous neuronal progenitor cell proliferation and migration (DCX+/Ki67+) that was evident in the hydrogels. These findings showed that self-assembling silk fibroin hydrogels effectively induce phenotypic changes in microglia and macrophages chronically after stroke that might favor tissue neurogenesis. These are important features for the development of next-generation stroke therapies.

## Graphical abstract

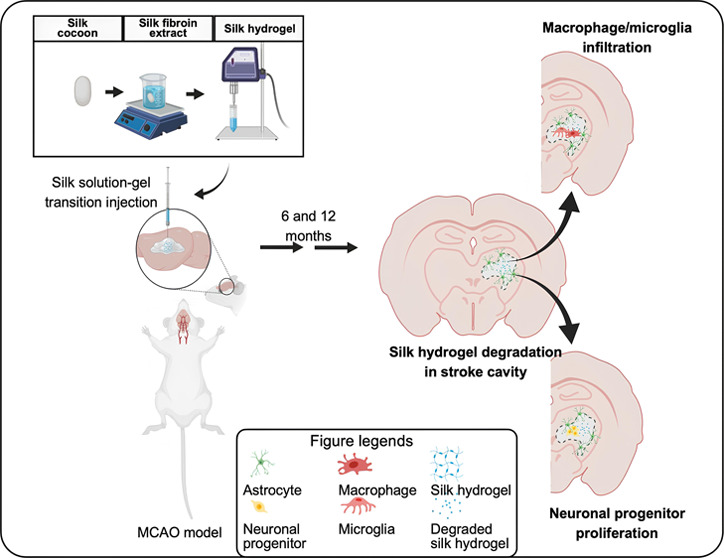

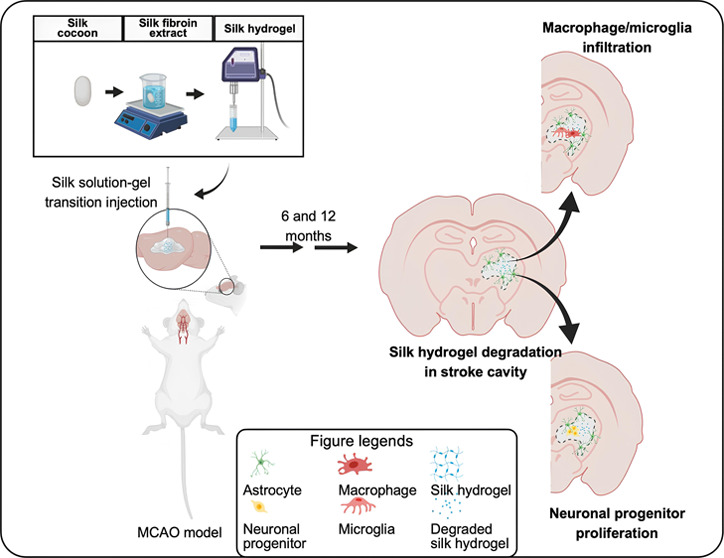

## Introduction

Stroke remains the most common cause of severe and long-term disability in adults worldwide.^1^ Revascularization reduces brain damage and loss of function,^2^ is the only approved therapy for ischemic stroke.^3^ However, provision to stroke patients is limited due to acute therapeutic time windows^4^ and no effective treatments are available that target the chronic phases of ischemic stroke. Thus, most patients who do not receive acute revascularization therapies show long-lasting neurological impairment.^5^

A major problem with cell-based therapies and biologics encountered in preclinical and clinical studies is the paucity of suitable delivery technologies that protect and retain therapeutic payloads in the stroke cavity.^6^ One solution to overcome this limitation is to use a biomaterial as a carrier.^7^ For example, stem cells coadministered during transplantation with a carrier can enhance stem cell viability, proliferation, and retention at the target site.^8^ Hydrogels are key contenders for this goal because they have the potential to (1) support cell behavior by presenting biomechanical and biochemical cues, (2) provide a fibrillary structure to support cells, and (3) furnish tunable mechanical and biochemical properties.^9^

Ischemic stroke is characterized by infiltration of large numbers of macrophage/microglia into the infarct area to promote clearance of necrotic cell debris, forming a stroke cavity.^10^ Injectable biomaterials into the stroke cavity offer an opportunity to offer a tissue-mimetic extracellular matrix and elicit endogenous brain tissue repair mechanisms^11^ for treatment of chronic ischemic stroke. However, the typical candidate materials have thus far failed in their translation to the clinic, mainly due to fast/slow degradation.^12^ Engineered biomaterials are believed to promote regeneration,^13^ but more knowledge on their biocompatibility and degradation over time after stroke is required.

Silk fibroin-based hydrogels have a historic track record of biocompatibility, excellent mechanical properties especially its native fiber form,^14^ and controllable biodegradation rates,^15^ tunable for soft (i.e., brain) and hard tissue engineering applications. The *in vivo* degradation rates of silk material formats can be altered from minutes to years by controlling the structure and morphology,^16^ are successfully used clinically in humans (e.g., Silk Voice injectable implant),^17^ but have not yet been tested in the chronic stroke setting.^18^ We and others have investigated the use of injectable silk fibroin hydrogels for poststroke brain repair^19,20^ because the externally triggered solution-to-gel transition enables minimally invasive stereotactic injection. Self-assembling silk fibroin hydrogels exhibit excellent conformal fit, without swelling due to their high-water content during solution-gel transition (80–98%), thereby lending itself well to intracerebral injection into the stroke cavity.^21^ We found that increasing fibroin concentration from 2% w/v to 4% w/v in self-assembling hydrogels manufactured by sonication accelerates β-sheet nanocrystalline network formation, resulting in an increase of the elastic modulus from ∼0.17 kPa at 2% to ∼1 kPa at 4%, providing mechanical strength similar to that of brain tissue.^21^ Though gelation viscosity increases with β-sheet formation, we have previously found that mechanics can be tuned independently of concentration through chemical or physical cross-linking so that silk hydrogels can be tailored with elastic versus viscoelastic mechanics while keeping the same 4% w/v fibroin content.^22^

*In vivo* silk fibroin degradation in part relies on host immune cells, specifically macrophages and foreign-body giant cells, and occurs via immune-cell-mediated pathways (i.e., phagocytosis and extracellular proteolytic degradation). In studies up to 2 months after stroke, silk-hydrogel treated animals showed that reduced microglial/macrophage response in the core of the lesion potentially increased angiogenesis with no visible signs of hydrogel degradation.^20^ However, the long-term fate of the silk fibroin hydrogel, as well as the tissue response to the hydrogel and its degradation, is currently unknown. Here, we show that acellular silk hydrogels can potentially support tissue reconstruction over a 12-month duration after a stroke by generating a prorepair environment within the hostile stroke cavity and activating endogenous repair processes, including neurogenesis.

## Materials and Methods

### Silk Fibroin Hydrogel Manufacture

Silk solution was prepared from *Bombyx mori* cocoons, as previously reported.^20^ In brief, the cocoons were degummed by boiling for 60 min in 25 mM Na_2_CO_3_. The degummed silk was dissolved in 9.3 M LiBr at 60 °C for 3 h and then dialyzed against water for 48 h (molecular weight cutoff 3500 Da), yielding a 5–6% w/v silk fibroin solution. Next, 10× phosphate buffered saline (PBS) was added to the silk fibroin solution to obtain physiological osmolarity for the final preparation. The resulting 4% w/v silk fibroin solution was filter-sterilized. Physically cross-linked silk hydrogels were manufactured by sonication with a digitally controlled probe sonicator (Sonoplus HD 2070, Bandelin, Berlin, Germany) fitted with a 23 cm long sonication tip (0.3 cm diameter tip and tapered over 8 cm) as previously described.^20,21^ A total volume of 4 mL of 4% w/v silk fibroin solution in water was placed on ice in 15 mL Falcon tubes (1.4 cm diameter and 11 cm long) (Greiner Bio-One GmbH, Kremsmünster, Austria) and exposed to 3 sonication cycles at 30% amplitude (one cycle consisted of 30 s on and 30 s off) to induce the solution–gel transition. The sonicated silk fibroin samples were then drawn into Hamilton syringes and injected into the animals.

### Middle Cerebral Artery Occlusion (MCAo)

Animal procedures were performed in accordance with the UK Animals (Scientific) Procedures Act (1986) and the Ethical Review Process of the Institute of Pharmacy and Biomedical Sciences of the University of Strathclyde, in adherence with ARRIVE guidelines.^23^ All animal procedures were approved by the Home Office of the United Kingdom (Project license number 60/4469). Male Sprague–Dawley rats (weight 240–290 g, 8–9 weeks old, Harlan, UK, *n* = 8) were maintained on a 12 h light/dark schedule, with food and water available *ad libitum*. For the right middle cerebral artery occlusion (MCAo) rat model of stroke, the animal was placed under isoflurane anesthesia (4% for induction, 2% for maintenance in 30% oxygen), and the body temperature was maintained at 37 ± 1 °C. A propylene filament (Doccol Corporation, USA, tip diameter with coating 0.33 ± 0.02 mm) was then advanced to the ostium of the MCA in the circle of Willis. The MCA was occluded for 1 h prior to reperfusion by retracting the filament to the common carotid bifurcation, mimicking the occlusion observed in two-thirds of patients undergoing ischemic stroke.^24^ After recovery from anesthesia, the animals were assessed for forelimb flexion and contralateral circling. Daily postoperative care and neurological assessment were performed until the animals recovered preoperative weight. A priori exclusion criteria were any animal not exhibiting signs of MCA occlusion (i.e., unilateral forelimb flexion) or any animal found to be moribund due to excessive weight loss (>20% of start weight).^25^ The severity of the stroke-induced deficits was established by monitoring animals using a neurological deficit grading scale of 0 to 4, where 0 = no observable deficit; 1 = forelimb flexion; 2 = decreased resistance to lateral push (and forelimb flexion) without circling; and 3 = decreased resistance to lateral push (and forelimb flexion) with circling. An additional score of 4 was added if the animal appeared unstable or exhibited reduced spontaneous motility.

### Stereotactic Surgery

Two weeks after MCAo, the rats were anesthetized with isoflurane (4% induction, 2% maintenance) and received 4% w/v self-assembling silk fibroin hydrogel (*n* = 8). No vehicle control group was used as this is a longitudinal study of hydrogel behavior over time. Animals were placed in a stereotactic frame, and injections (10 μL, at a rate of 2 μL/min) were performed at coordinates −1.5 mm from midline (M-L axis), −3.5 mm (A-P axis) from Bregma, and −6.5 mm ventral to the skull surface (D-V axis) using a 10 μL Hamilton syringe with a 22G blunt-tip needle.

### Perfusion-Fixation of Tissue

Animals were randomly allocated to be terminated at 6- and 12-months postimplantation using Research Randomizer (https://www.randomizer.org/) (*n* = 4/group) for analysis of the *in situ* distribution of the silk hydrogels and cell infiltration within the hydrogel (Figure 1A). Animals underwent transcardial perfusion with 0.9% normal saline followed by 4% ice-cold paraformaldehyde in 0.2 M PBS. Brains were postfixed in 4% paraformaldehyde for 24 h prior to cryopreservation in 30% sucrose in PBS with 0.01% sodium azide for 72 h at 4 °C. Histologic sections (30 μm thickness) were coronally cut on a cryostat (Leica CM1850, UK) and placed directly onto microscopic slides to preserve tissue morphology. For all analyses, the investigator was blinded to the timing poststroke by an independent investigator.

**Figure 1:**
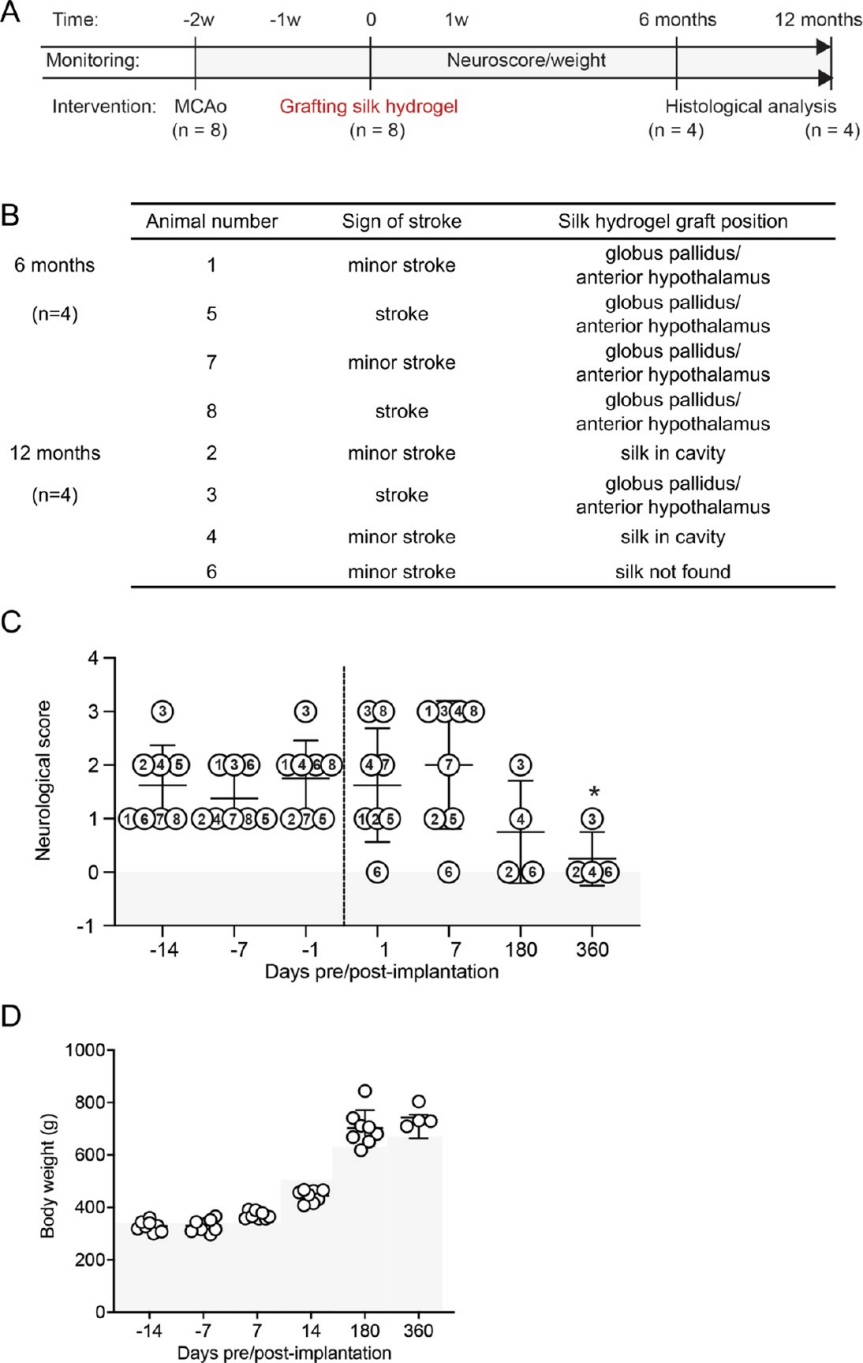
Neurological function and body weight were unaffected by silk fibroin hydrogel implants. (A) Experimental timeline, stroke intervention, and assessment. Right transient middle cerebral artery occlusion (MCAo) was performed on 8 rats at 2 weeks prior to the grafting surgery. At time 0, animals were given stereotactic intracerebral injections of 4% w/v self-assembling silk hydrogels. Hydrogels were prepared by sonication, and stereotactic injection into the stroke cavity was performed during the sol–gel transition. Animals were randomly assigned to be terminated at 6- and 12-months postimplantation. (B) Stroke lesions were found in all animals after MCAo, indicating minor or striatal stroke. (C) Neurological score was assessed over 2 weeks during post-MCAo recovery (day – 14 = after 24 h, day – 7 = after 7 days, and day – 1 = after 14 days) and again after grafting at 24 h, 1 week, 6 months, and 12 months. Numbers in plot symbols corresponds to animal identification number. (* *p* < 0.05 versus 1-week postgrafting), and (D) body weight was determined over 2 weeks during post-MCAo recovery (day – 14 = after 24 h, day – 7 = after 7 days, and day – 1 = after 14 days) and again after grafting at 1 week, 2 weeks, 6 months, and 12 months. Gray shading indicates the standard growth curve of Sprague–Dawley rats at the corresponding time points. Data were presented as mean ± SEM.

### Hematoxylin and Eosin (H&E) and Masson Trichrome Staining

The tissues were stained with hematoxylin and eosin to identify the lesion and graft localization in whole brain tissue. Masson trichrome staining was performed to visualize the gross morphology of the silk hydrogel implants within the cavity according to the manufacturer’s protocol (ab 150686, Abcam, UK). The stained sections were viewed and photographed with a bright field microscope.

### Immunohistochemistry

Brain sections were washed three times for 5 min in PBS, followed by 40 min of permeabilization in 10% v/v blocking sera and in PBS with 0.3% v/v Triton X-100 (Sigma) at room temperature. Primary antibodies were diluted in PBS with 10% v/v normal serum and 0.3% v/v Triton X-100 and applied to the sections, followed by incubation at 4 °C overnight. Phenotypic markers consisted of rabbit antiglial fibrillary acid protein (GFAP) (1:1000, Z0334, DAKO, CA, USA) to visualize the glial scar; rat anti-CD11b (1:200, ab1211, Abcam, UK) to detect microglia/macrophages; rabbit anti-CD86 (1:100, ab269587, Abcam, UK) to visualize immune-activated microglia/macrophages; mouse anti-CD206 (1:50, sc376108, Santa Cruz Biotechnology, Texas, USA) to detect a shift toward a phagocytic and tissue-remodeling phenotype; rabbit anti-Ki67 (1:500, ab15580, Abcam, UK) to visualize proliferating cells; and chicken anti-doublecortin (DCX) (1:150, ab153668, Abcam, UK) to visualize neural progenitor cells. The unreacted primary antibodies were removed from the sections by three 5 min rinses in PBS, and appropriate secondary Alexa Fluor 488 or Alexa Fluor 555 antibodies (1:500, Invitrogen, UK) were applied for 2 h at room temperature, followed by three 5 min PBS washes. The sections were coverslipped with DAPI containing Vectashield (Vector Laboratories, UK) and stored at 4 °C prior to imaging. Images were captured and analyzed using WinFluor V3.9.1 (Nikon Eclipse E600).

### Statistical Analyses

Animal weight and neurological deficit data were shown as individual animal data points and expressed as mean values ± standard error of the mean (SEM). Statistical analysis were performed using one-way ANOVA followed by Dunnett’s multiple comparison post hoc test (Prism 10.0; GraphPad Software Inc., USA, CA). A *P* value of *P* < 0.05 was considered significant.

## Results

### Neurological Recovery and the Silk Hydrogel–Host Tissue Interface

Ischemic stroke in the caudate putamen (striatum) was successfully created by MCAo in all animals (*n* = 8) with no animals excluded (Figure 1B). After grafting, 1 rat showed full restoration of its neurological functions, with no observable deficit (score = 0), in line with previous work^26^ and our own^20^ showing full restoration in some animals within 14 days post-MCAo, whereas the other 7 animals showed partial recovery (those with striatal stroke) (score = 1–3) (Figure 1C). The highest neurologic deficit was observed at 1-week postimplant. After 12 months postgrafting, the neurological score was significantly decreased and approached the original baseline levels in all animals (Figure 1C). No significant body weight loss was observed during the study period following the hydrogel grafting, as animal body weights were comparable to the standard growth curve for Sprague–Dawley rats at relative time points^27^ (Figure 1D).

Injection of silk fibroin into the lesion cavity resulted in a robust *in situ* gelation with a good interface formed between the hydrogels and the host tissue. Silk hydrogel remnants were still present, although with visibly reduced material integrity, at 12 months postgrafting (Figures 2 and 3). The presence of silk hydrogel was indicated by a light pink color after hematoxylin and eosin staining and a purple color after trichrome staining. Small gaps were observed and could potentially be artifacts that arose during sample preparation.

**Figure 2:**
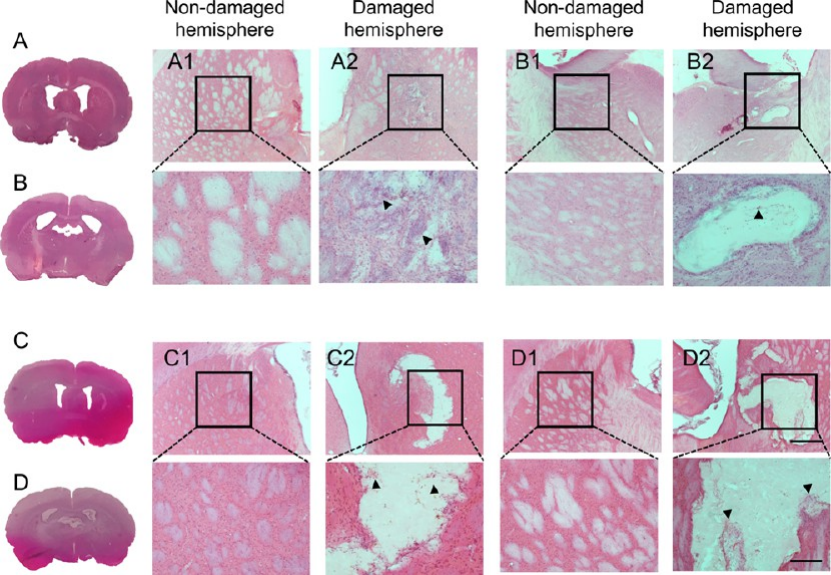
Endogenous cells present in the silk hydrogel graft. Representative hematoxylin and eosin-stained coronal brain sections at (A, C) the level of the globus pallidus and (B, D) the level of the anterior hypothalamus at (A, B) 6 or (C, D) 12 months after transplantation with self-assembling silk fibroin hydrogels (dotted line represents higher magnification, below panels). The whole brain sections and magnified figures illustrate (A1, B1, C1, D1) the nondamaged hemisphere and (A2, B2, C2, D2) the damaged hemisphere. Representative image showing the presence of the silk fibroin hydrogel graft in the striatal lesion surrounded by endogenous invading cells (nuclei showing purple hematoxylin staining; arrow). Silk hydrogel is indicated by light pink staining. Scale bars: 200 μm; zoom 50 μm.

**Figure 3:**
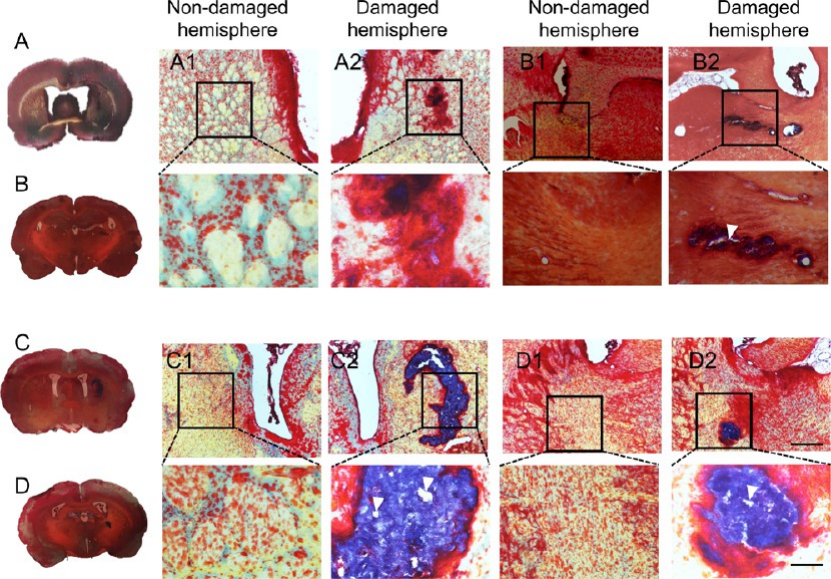
The silk hydrogel graft deposited over time. Representative trichrome stained images of coronal brain sections at (A, C) the level of the globus pallidus and (B, D) the level of the anterior hypothalamus at (A, B) 6 or (C, D) 12 months after transplantation with self-assembling silk fibroin hydrogels (dotted line represents higher magnification, below panels). The whole brain sections and magnified figures illustrate (A1, B1, C1, D1) the nondamaged hemisphere and (A2, B2, C2, D2) the damaged hemisphere. Representative image showing the presence of a good space confirming silk hydrogel deposits in the stroke lesion. Signs of hydrogel degradation were observed (i.e., looser structure; arrow). Silk hydrogel is indicated by purple staining (blue/purple = collagen/silk fibroin; red = cytoplasm; black/blue = nucleus). Scale bars: 200 μm; zoom 50 μm.

### Silk Hydrogels-Macrophage and Astrocyte Infiltration

At 6 months postgrafting, CD11b+ macrophage/microglia were found in abundance around the small silk fibroin hydrogel remnants that were spread across the stroke cavity; however, only a very few cells invaded the hydrogel itself (Figure 4). By 12 months, a marked increase in macrophage density within the hydrogels was observed compared with the 6-month time point, and the infiltrating macrophage/microglia were located close to astrocytes (Figure 5).

**Figure 4:**
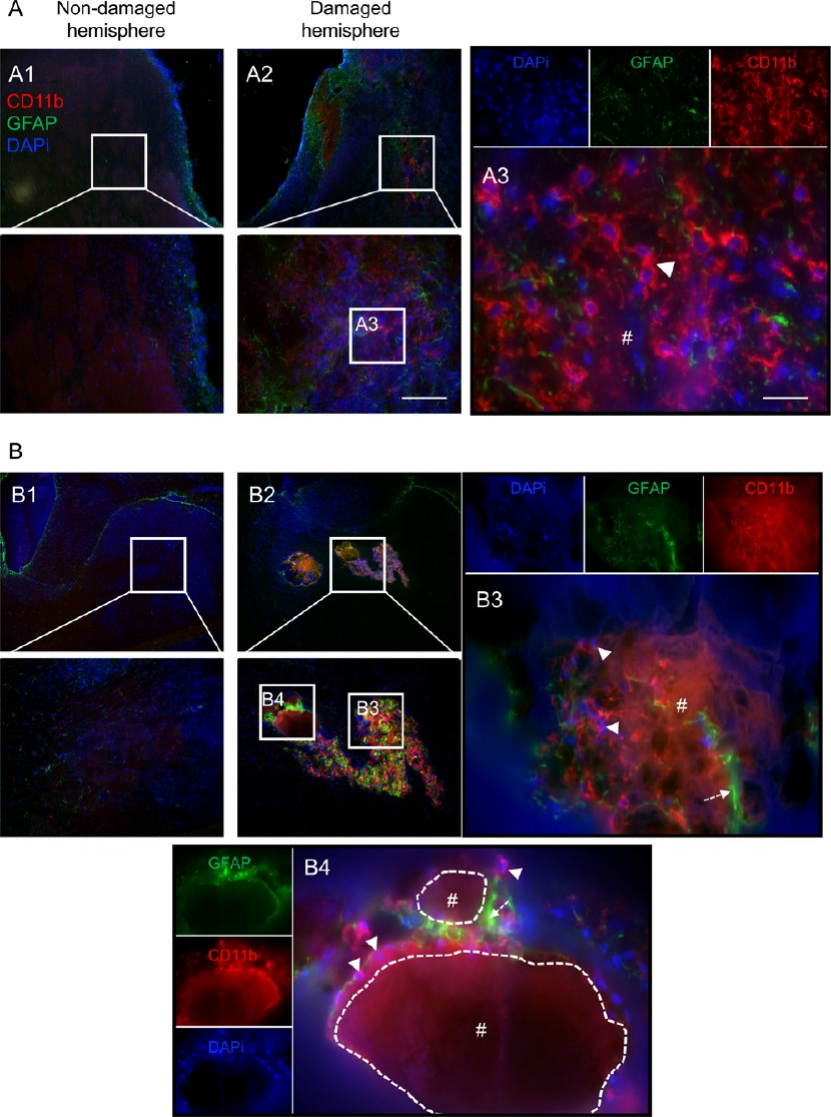
Silk hydrogels promoted microglia/macrophage and astrocyte infiltration at 6 months postgrafting. Representative CD11b+ (red)/GFAP+ (green)/DAPI (nuclei in blue) staining images of coronal brain sections at (A) the level of the globus pallidus and (B) the level of the anterior hypothalamus at 6 months postgrafting with self-assembling silk fibroin hydrogels (dotted line represents higher magnification, below panels). The whole brain sections and magnified figures illustrate (A1, B1) the nondamaged hemisphere and (A2–A3 and B2–B4) the damaged hemisphere. Representative image showing CD11b+ microglia/macrophages (arrow) and GFAP+ astrocytes (dotted arrow) surrounding a grafted hydrogel (hash symbol with white dotted outline). Scale bars: 200 μm; zoom, 20 μm.

**Figure 5:**
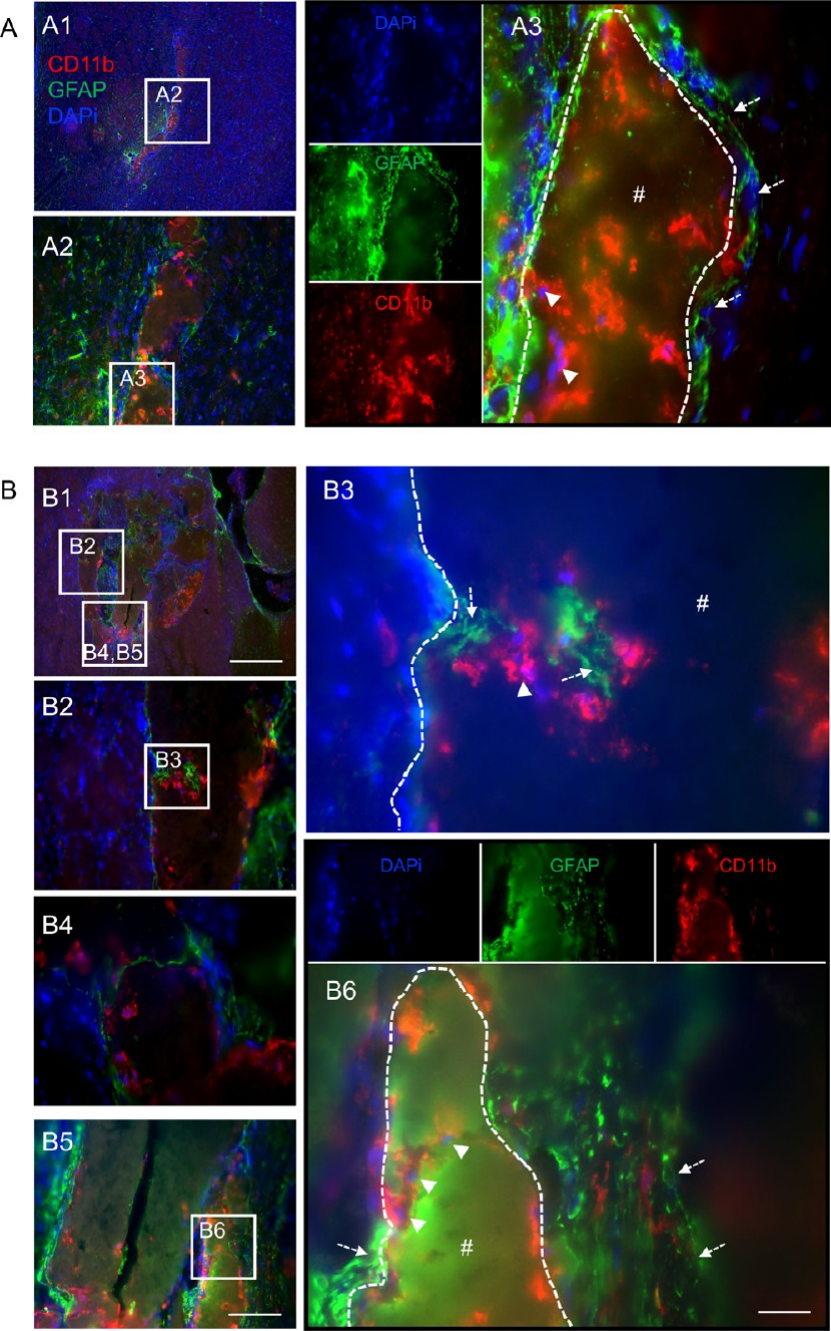
Silk hydrogel promoted microglia/macrophage and astrocyte infiltration at 12 months postgrafting. Representative CD11b+ (red)/GFAP+ (green)/DAPI (nuclei in blue) staining images of coronal brain sections at (A) the level of the globus pallidus and (B) the level of the anterior hypothalamus at 12 months postgrafting with self-assembling silk fibroin hydrogels. The whole brain sections and magnified figures illustrate (A1–A3, B1–B6) the damaged hemisphere. Representative image showing CD11b+ microglia/macrophages (arrow) and GFAP+ astrocytes (dotted arrow) at the peri-infarct and infarct areas of a grafted hydrogel (hash symbol with white dotted outline). Scale bars: 200 μm; zoom 50 μm; inset 20 μm.

Degradation of the silk hydrogels could be a key factor that enabled host cell infiltration and brain tissue reconstruction. Some cases showed invasion by microglia and macrophages, which are necessary for both tissue clearing and silk fibroin hydrogel degradation. Given the limitations of the M1/M2 terminology, we distinguished the phenotypes of microglia/macrophages based on marker expression: CD86 expression reflecting immune-interactive functions and CD206 expression commonly linked to tissue remodeling and debris clearance. At 6 months postgrafting, CD206-expressing microglia/macrophages dominated in the hydrogels (Figure 6). By contrast, macrophages coexpressing the CD86 and CD206 were the most common cell phenotypes infiltrating silk fibroin hydrogels at 12 months (Figure 7).

**Figure 6:**
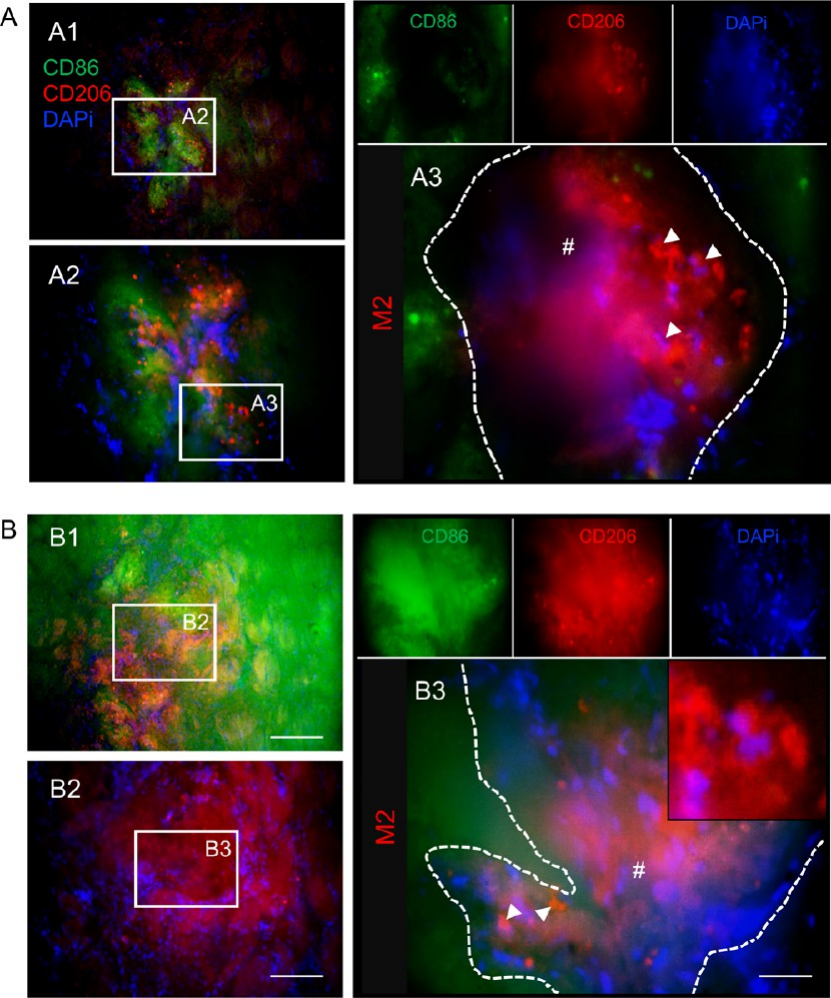
Silk hydrogels promoted CD206 expressing macrophage infiltration at 6 months postgrafting. Representative CD86 (green)/CD206 (red)/DAPI (nuclei in blue) stained images of coronal brain sections at (A) the level of the globus pallidus and (B) the level of the anterior hypothalamus at 6 months postgrafting with self-assembling silk fibroin hydrogels. The whole brain sections and magnified figures illustrate (A2–A3 and B1–B3) the damaged hemisphere. Representative image showing CD206+ macrophages (arrows) in the infarct area of the grafted hydrogels (hash symbol with white dotted outline). Scale bars: 200 μm; zoom 50 μm; inset 20 μm.

**Figure 7:**
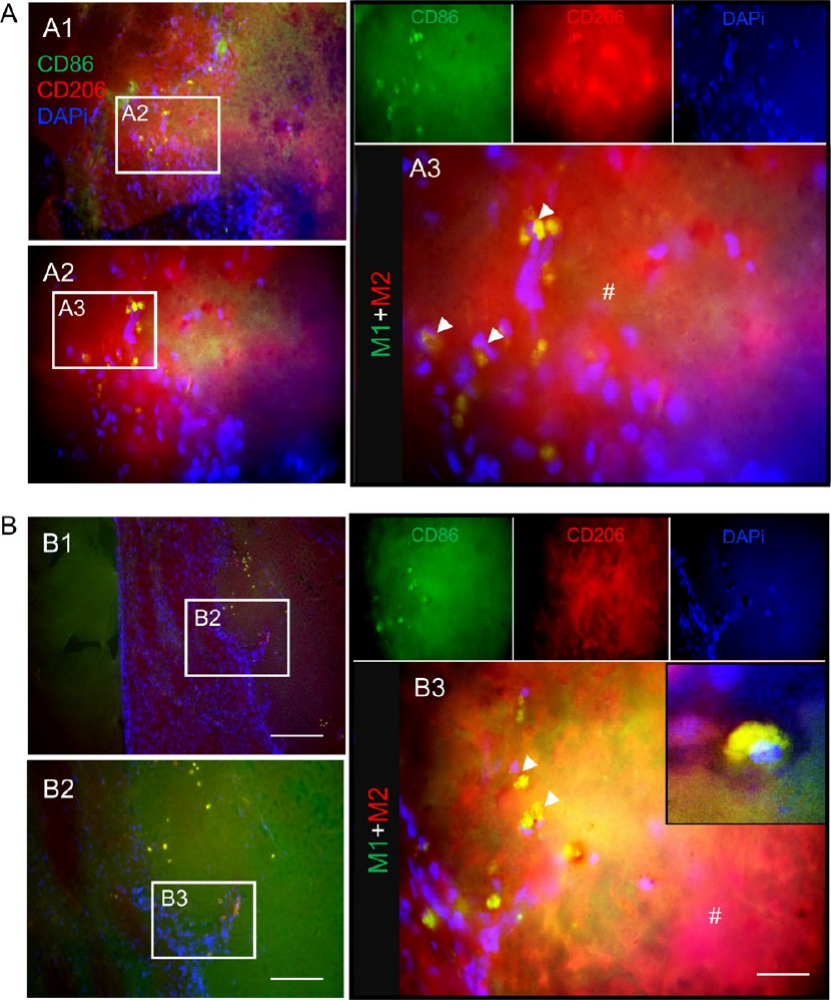
Silk hydrogel promoted hybrid CD86 expressing and CD206 expressing macrophage infiltration at 12 months after grafting. Representative CD86 (green)/CD206 (red)/DAPI (nuclei in blue) stained images of coronal brain sections at (A) the level of the globus pallidus and (B) the level of the anterior hypothalamus at 12 months postgrafting with self-assembling silk fibroin hydrogels. The whole brain sections and magnified figures illustrate (A2–A3 and B1–B3) the damaged hemisphere. Representative image showing hybrid CD86+/CD206+ cells (arrows) in the infarct area of the grafted hydrogels (hash symbols). Scale bars: 200 μm; zoom 50 μm; inset 20 μm.

### Silk Hydrogels-Neuronal Progenitor Cell Infiltration

We observed cells within the hydrogels that did not show staining for macrophages or microglia and astrocytes. In the brain, neural progenitor cell (NPC) migration toward the damaged tissue is a spontaneous endogenous poststroke response that elicits tissue remodeling and new brain tissue formation. We therefore determined whether our material enhanced neuronal NPC proliferation and migration to lesion sites by examining doublecortin (DCX) and Ki67 as NPC and proliferating cell markers, respectively. Double positive DCX and Ki67 cells, which were considered proliferating NPCs, were found both in the peri-infarct area and in the infarct area of the hydrogels (Figure 8).

**Figure 8:**
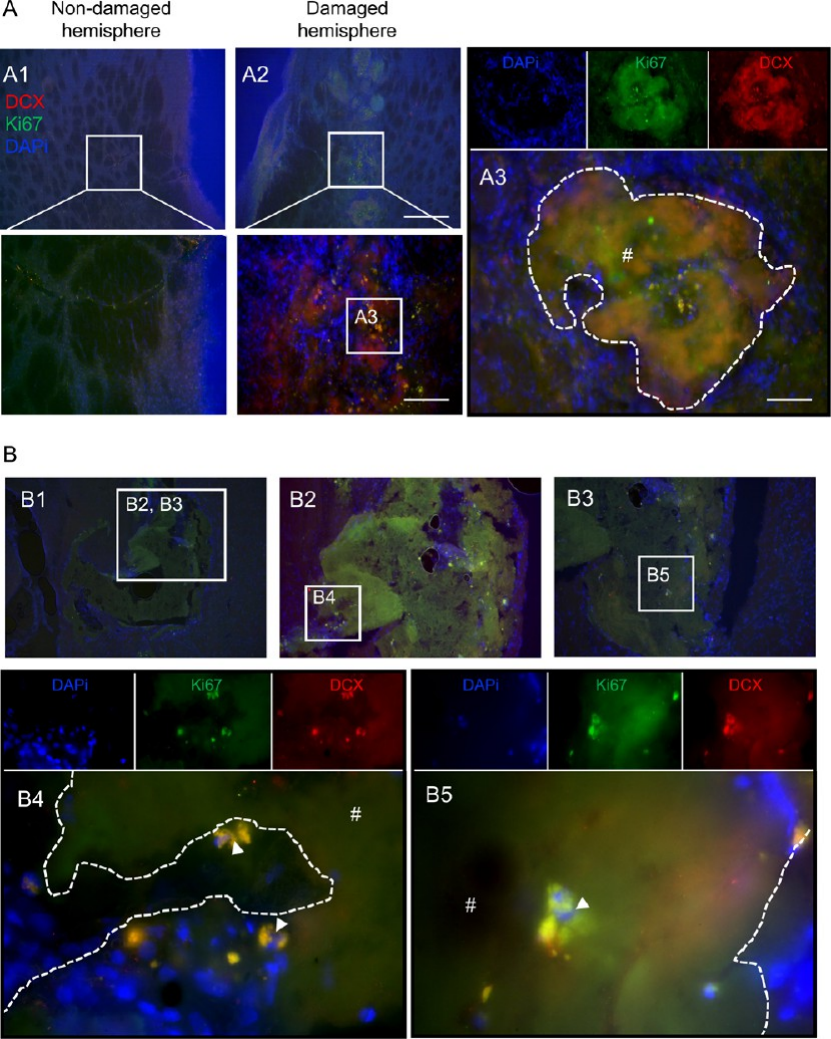
Neuronal progenitor cells invaded into silk hydrogels at 6- and 12-months postgrafting. Representative DCX+ (red)/Ki67+ (green)/DAPI (nuclei in blue) stained images of coronal brain sections at (A) 6 months and (B) 12 months postgrafting with self-assembling silk fibroin hydrogels (dotted line represents higher magnification, below panels). The whole brain sections and magnified figures illustrate (A1) the nondamaged hemisphere and (A2–A3 and B1–B5) the damaged hemisphere. Representative image showing proliferating neuronal cells (DCX+/Ki67+ cells) (arrow) in the peri-infarct and infarct areas of the grafted hydrogels (hash symbol with white dotted outline). Notably, a greater number of proliferating neuronal cells were observed within the hydrogels at 12 months postgrafting. Scale bars: 200 μm; zoom 50 μm; inset 20 μm.

## Discussion

In the search for better stroke therapies, injectable hydrogels are promising contenders in minimally invasive therapeutic interventions, especially in the context of cell therapies.^28^ The advantage of self-assembled silk hydrogels is that their solution–gel transition enables their administration through a thin needle to the lesion site and subsequent *in situ* gelation. This ultimately provides structural support but without swelling and tissue-like mechanics.^29^ In addition, stroke infarcts are typically irregular in shape; therefore, the use of shape-adaptable hydrogels allows the filling of irregular defects, thereby facilitating good host tissue integration.^10^

In this study, we examined the long-term biocompatibility and degradation of silk hydrogels in the stroke microenvironment by monitoring *in vivo* hydrogel biodegradation over 12 months. The intraluminal thread model creates a complex array of microenvironments, with cavitation representing the most severe form of damage.^30^ The histology of silk fibroin hydrogel-treated brains elicits the promotion of astrocyte (GFAP+) and microglia/macrophage (CD11b+) infiltration into the hydrogels rather than the promotion of astrocytic scar formation surrounding implanted areas. At 6 months postgrafting, the majority of the invading cells were found at the tissue/hydrogel boundary and typically were CD206 expressing microglia/macrophages commonly linked to tissue remodeling and debris clearance. By contrast, at 12 months postgrafting, a substantial infiltration was observed in the center of the hydrogels by macrophages that expressed CD206 and CD86, indicating immune-activated cells. The areas surrounded by macrophages showed evidence of hydrogel degradation with the degraded sites potentially providing a niche for endogenous neuronal progenitor cell proliferation and migration (DCX+/Ki67+). The glial scar creates a physical barrier that seals off the stroked area as a way to limit damage and tissue cavitation in adjacent areas.^30^ However, this scarring also impedes tissue regeneration by limiting access to appropriate immune cells while obstructing neuronal ingrowth.^31^ Our present study demonstrates that silk fibroin hydrogels are a suitable biomaterial for promoting tissue repair, as evidenced by enhanced infiltration of astrocytes (GFAP^+^), microglia/macrophages (CD11b^+^), and proliferating neural progenitor cells (DCX^+^/Ki67^+^) into the hydrogels, indicating that residual scarring did not completely limit host cell recruitment into the hydrogels. Similar observations have been made with other degradable materials, including IKVAV peptides^32^ and poly­(lactic-*co*-glycolic acid)-modified hyaluronic acid.^33^ Both structural support and inductive cues are necessary for cells to migrate into the hydrogel. Hydrogels provide a structure that supports both cell infiltration and ingrowth.^34,35^ However, achieving suitable mechanical properties for the hydrogels and their sufficient tissue residence time is difficult without resorting to hydrogel cross-linking. The available cross-linker materials are often cytotoxic, so they can adversely affect biocompatibility.^36^ Here, the use of physical, rather than chemical, cross-linking of the silk hydrogels eliminated the need for these harsh chemicals. We used 4% w/v silk hydrogels that have a 1 kPa stiffness and a reliable solution–gel transition,^21^ and our processed silk material achieved a mechanical stiffness matching that of brain tissue.^21^ This work also complements our earlier studies that examined the performance of these hydrogels during the first 7 weeks post stroke.^20^ Self-assembled 4% w/v silk hydrogels are viscoelastic,^22^ making them particularly suitable for neural tissues.^37^

In the present study, injectable silk hydrogels evoked the colocalization of microglia/macrophages with infiltrating astrocytes. This mobilization of microglia/macrophages might be followed by a “chain-like” migration of pioneering astrocytes. In line with the expected acute tissue response, we have previously shown that macrophage infiltration is also a typical foreign-body response toward silk fibroin hydrogels.^20^ How these innate immune cells guide astrocyte migration into the damaged area or the exact sequence of events remains to be elucidated. We speculate that “invasion trails” likely arise due to a combination of cell–cell signaling by invading immune cells^38^ and changes in the conductive mechanical properties. For example, the internal architecture of a hydrogel, such as its porosity and topographical cues, offers potential mechanisms for modulation of nutrient/trophic factor diffusion and cell motility—factors that ultimately affect the development of neural progenitors.^39^ We know that self-assembling silk hydrogels are viscoelastic, with various impacts on cell biology factors,^22^ including phenotype, trophic factor release, and possible migration behavior. The injectable silk fibroin hydrogels used here were conducive to the formation of new tissue through recruitment and proliferation. We speculate that innate immune cells ultimately recruit other brain cells that gradually repopulate the hydrogel material. However, more work is required to understand the molecular mechanisms and functional consequences.

Macrophage density within the hydrogel and particularly the density of the CD206-expressing microglia/macrophages are key to promoting rapid hydrogel biodegradation, suggesting that cells were able to better access the implanted hydrogels following degradation. Materials promoting *de novo* tissue formation must be biodegradable, as the invading host cells must be able to remodel and degrade the biomaterial to facilitate the formation of a new neuronal network.^40^ Infiltrating macrophages are critical for the initiation of hydrogel material degradation. The CD86-expressing macrophages are associated with a foreign-body response, while the CD206-expressing phenotype is thought to be crucial for attracting cells from the host tissues to repopulate the tissue damage site and to replace the implanted material with new tissue.^24,41^ Upregulation of matrix metalloproteinases-2 and -9 in macrophages after stroke is responsible for the degradation of the native extracellular matrix, the breakdown of the blood brain barrier, and the increase of immune cell invasion at the injury site.^42^ This tissue response potentially triggers further macrophage/microglia recruitment into the implanted materials, leading to material remodeling and degradation.^43^ We observed phenotypic changes in macrophages over time: the CD206 expressing macrophage response dominated at 6 months postimplantation, indicating that this could promote brain tissue remodeling during stroke recovery, but both CD86+ and CD206+ phenotypes occurred after 12 months. We speculate that this shift is mediated by signaling factors arising from the silk hydrogel and its degradation products. A similar shift has been observed in response to the long-term degradation of porcine-derived urinary bladder extracellular matrix hydrogels. The matrix in that study showed an approximately 80% degradation of the low-strength area within 14 days, and 32% of the high-intensity area was degraded within 3 months. The material resorption profile was matched by glial cell activity and a phenotypic macrophage shift from an M1-like to an M2-like state.^24^

The current paradigm is to match hydrogel degradation with tissue remodeling, ultimately resulting in material replacement (i.e., degradation) with newly formed tissue.^44^ A mismatch typically results in a failure to guide proliferation and differentiation of local progenitor cells. For example, soft collagen hydrogels often degrade faster than desired,^45^ and they pose an additional theoretical risk of introducing prion diseases into the brain. Hyaluronic acid hydrogels cause toxicity to parenchyma host tissues, leading to accelerated aging and demyelinating diseases.^46,47^ Studies on the long-term performance of silk in healthy and pathological brains are scarce. This is the first study to report the long-term performance of silk in a stroked brain. However, silk hydrogel performance and degradation have been studied in other soft and hard tissues.^48^ Both silk secondary structure and format are known to modulate silk degradation and, ultimately, tissue performance, and several studies have reported improved *in vivo* tissue repair in response to silk.^15,49^ For example, intramyocardially injected silk hydrogels (2% w/v) were completely degraded within 1 month and prevented negative left ventricle remodeling,^50^ whereas physically cross-linked silk hydrogels (2% w/v) in a rat femoral segmental defect model were degraded within 3 months and promoted bone regeneration.^51^

Ultrasound imaging has also been used to track material degradation in subcutaneous or muscle implant models rather than intracranial models,^52,53^ where resolution for small hydrogel implants may be limiting and therefore histology remains the applicable technique for measurement for brain implants,^20,54^ and noninvasive imaging has been applied to monitor the innate immune response.^18^ However, little is known about silk hydrogel performance in the immune-privileged central nervous system. Implantation of self-assembling silk hydrogels (2% w/v) containing mesenchymal stem cells showed a 50% material reduction after 1 month in the absence of an inflammatory response.^54^ Our present study showed that self-assembling silk hydrogels were slowly degraded in the chronic stroke brain, with a substantial silk material loss occurring over 12 months. Silk degradation did not evoke any overt adverse tissue response, and our data suggest a mild acute response that declined further over time.

We also examined the impact of silk hydrogels on endogenous neuronal cells. Neural progenitor cells are promising for stroke tissue repair, because these cells have the potential to generate all neural cell types present in the brain. Stroke triggers neural progenitor cell proliferation in the subventricular zone.^55^ However, these neural progenitor cells cannot reach the stroke site; one barrier is the glial scar.^56^ Therefore, materials that can suppress glial scar formation while also supporting neural progenitor cell migration into the stroke area are promising, because this harnesses the endogenous repair mechanisms that ultimately contribute to tissue regeneration. *In vitro* studies indicate that silk is able to protect hiPSC-derived neurons and glial cells in long-term (2-year) cultures,^57^ suggesting that this supportive microenvironment might also be present *in vivo*. In the present study, neural progenitor cells were able to migrate along the anterior and posterior lateral ventricles toward the damaged tissues. We also observed neural-like cell proliferation and infiltration within the silk hydrogel, indicating that the proliferation of these migrating neural progenitors could promote brain tissue repair after stroke. This observation is exciting, because it suggests that silk hydrogel placement turned the stroke microenvironment into a hospitable microenvironment.

The cellular mechanism(s) underlying these microenvironment changes remains to be elucidated, but newly formed blood vessels or direct infiltration through the surrounding parenchyma host tissue is potentially involved. This scenario is plausible because endothelial cell survival, growth, and microvascular network formation were already evident at 2 months post stroke.^20^ Similar findings have been reported for porcine-derived urinary bladder extracellular matrix hydrogels.^58^ Clearly, cells are required for improving functional outcomes in stroke. Endogenous cell recruitment is desirable to reduce the treatment complexity, but its effects are likely to be finite. Therefore, exogenous cell applications are important. For example, application of mesenchymal stem cells to acute stroke lesions using self-assembling silk hydrogels (2% w/v) improved functional recovery and restitution of stroke-damaged neuronal circuitry.^19^

## Conclusions

We demonstrate that injection of silk hydrogels into a stroke lesion resulted in the retention of the silk hydrogel within the lesion cavity in the chronic setting with excellent host tissue integration. Evidence of silk hydrogel degradation, alongside invading macrophages, neural progenitor cells, and astrocytes, was evident at 6 months and was more widespread at 12 months. Our findings show that the applied silk hydrogel triggered tissue remodeling, possibly promoted by CD206 expressing M2-like macrophages.
